# A Confidence Habitats Methodology in MR Quantitative Diffusion for the Classification of Neuroblastic Tumors

**DOI:** 10.3390/cancers12123858

**Published:** 2020-12-21

**Authors:** Leonor Cerdá Alberich, Cinta Sangüesa Nebot, Angel Alberich-Bayarri, José Miguel Carot Sierra, Blanca Martínez de las Heras, Diana Veiga Canuto, Adela Cañete, Luis Martí-Bonmatí

**Affiliations:** 1 Grupo de Investigación Biomédica en Imagen, Instituto de Investigación Sanitaria La Fe, Avenida Fernando Abril Martorell, 106 Torre A 7planta, 46026 Valencia, Spain; marti_lui@gva.es; 2 Área Clínica de Imagen Médica, Hospital Universitario y Politécnico La Fe, Avenida Fernando Abril Martorell, 106 Torre A 7planta, 46026 Valencia, Spain; sanguesa_cin@gva.es (C.S.N.); veiga_dia@gva.es (D.V.C.); 3 Quantitative Imaging Biomarkers in Medicine, QUIBIM SL. Edificio Europa, Av. d’Aragó, 30, Planta 12, 46021 Valencia, Spain; angel@quibim.com; 4 Departamento de Estadística e Investigación Operativa Aplicadas y Calidad, Universitat Politècnica de València, Camí de Vera s/n, 46022 Valencia, Spain; jcarot@eio.upv.es; 5 Unidad de Oncohematología Pediátrica, Hospital Universitario y Politécnico La Fe, Avenida Fernando Abril Martorell, 106 Torre A 7planta, 46026 Valencia, Spain; martinez_bladel@gva.es (B.M.d.l.H.); canyete_ade@gva.es (A.C.)

**Keywords:** tumor clustered habitats, confidence maps, data smearing, uncertainty exclusion, reproducible imaging biomarkers

## Abstract

**Simple Summary:**

There is growing interest in applying quantitative diffusion techniques to magnetic resonance imaging for cancer diagnosis and treatment. These measurements are used as a surrogate marker of tumor cellularity and aggressiveness, although there may be factors that introduce some bias to these approaches. Thus, we explored a novel methodology based on confidence habitats and voxel uncertainty to improve the power of the apparent diffusion coefficient to discriminate between benign and malignant neuroblastic tumor profiles in children. We were able to show this offered an improved sensitivity and negative predictive value relative to standard voxel-based methodologies.

**Abstract:**

Background/Aim: In recent years, the apparent diffusion coefficient (ADC) has been used in many oncology applications as a surrogate marker of tumor cellularity and aggressiveness, although several factors may introduce bias when calculating this coefficient. The goal of this study was to develop a novel methodology (Fit-Cluster-Fit) based on confidence habitats that could be applied to quantitative diffusion-weighted magnetic resonance images (DWIs) to enhance the power of ADC values to discriminate between benign and malignant neuroblastic tumor profiles in children. Methods: Histogram analysis and clustering-based algorithms were applied to DWIs from 33 patients to perform tumor voxel discrimination into two classes. Voxel uncertainties were quantified and incorporated to obtain a more reproducible and meaningful estimate of ADC values within a tumor habitat. Computational experiments were performed by smearing the ADC values in order to obtain confidence maps that help identify and remove noise from low-quality voxels within high-signal clustered regions. The proposed Fit-Cluster-Fit methodology was compared with two other methods: conventional voxel-based and a cluster-based strategy. Results: The cluster-based and Fit-Cluster-Fit models successfully differentiated benign and malignant neuroblastic tumor profiles when using values from the lower ADC habitat. In particular, the best sensitivity (91%) and specificity (89%) of all the combinations and methods explored was achieved by removing uncertainties at a 70% confidence threshold, improving standard voxel-based sensitivity and negative predictive values by 4% and 10%, respectively. Conclusions: The Fit-Cluster-Fit method improves the performance of imaging biomarkers in classifying pediatric solid tumor cancers and it can probably be adapted to dynamic signal evaluation for any tumor.

## 1. Introduction

**Figure 1:**
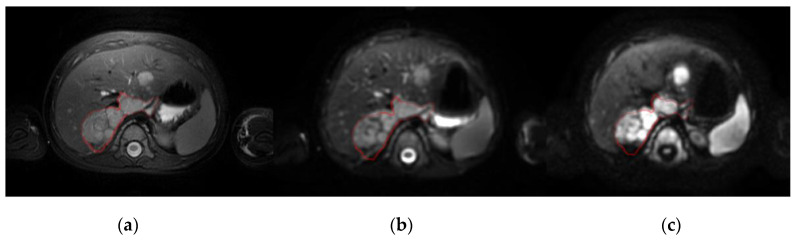
Baseline MRI of a patient with neuroblastoma in the right adrenal gland and liver metastasis shown on: (**a**), a T2-weighted image; (**b**), a DW image with b = 0 s/mm^2^; and (**c**), a DW image with b = 800 s/mm^2^ (right). The red line defines the primary tumor.

Although neuroblastic tumors are rare lesions, they are the most frequent extracranial solid cancer in children, and they include neuroblastoma, nodular ganglioneuroblastoma, intermixed ganglioneuroblastoma and ganglioneuroma [1]. In the main, tumor diagnosis, prognosis and monitoring decisions are initially based on information obtained from magnetic resonance (MR) and Metaiodobenzylguanidine scintigraphy (mIBG) images. Diffusion-weighted imaging (DWI) is a standard-of-care MR technique that enables the microscopic movement of water molecules in the extracellular space of biological tissues to be visualized by calculating the apparent diffusion coefficient (ADC) maps. In children with neuroblastic tumors DW images are usually obtained to better stage primary tumors, evaluating the local invasion and distant metastases (Figure 1). By using at least two *b*-values, the ADC can be calculated following an exponential signal decay model according to the equation ADC = ln(SI_1_/SI_2_)/(*b*_2−_*b*_1_), where SI_1_ and SI_2_ are the DWI signal intensity with different gradient factors of *b*_1_ and *b*_2_, which reflect the strength and timing of the gradients. The ADC values calculated have traditionally been used to estimate the potential malignancy of a lesion [2]. In clinical practice, most MR derived dynamic parameters, like the exponential ADC calculation, are extracted following a voxel-by-voxel approach in combination with a histogram analysis method over a region of interest (ROI), usually the whole tumor, such as the region average [3], minimum/maximum values [4] or percentile, skewness or kurtosis [5].

Although it may be considered in many oncology applications as a surrogate measure of cellularity and aggressiveness, ADC calculation may be biased by several factors. Some of the most relevant issues that influence the data quality and values are the signal-to-noise ratios in the reconstructed images, the *b*-values used in the sequence acquisition, the phase errors of eddy currents, and the geometric distortion associated to the echo-planar imaging (EPI) factor. In addition, selecting a voxel-based or a region-based signal decay analysis may affect the ADC values obtained [6], particularly as the use of average values may inaccurately represent a tumor or region of heterogeneous internal composition. The different source region definition adds further dispersion to the ADC values obtained at distinct centers and with different MR machines [7].

To improve the robustness of ADC measurements with respect to a voxel-based analysis, it may be useful to previously detect homogeneous tumor habitats as clusters. Identifying robust voxels may also help to standardize the measurement by maximizing signal and minimizing noise prior to extracting the ADC values. By adding maps of confidence habitats as regions in which stable ADC quantification is guaranteed, signal analysis biases will be minimized. We developed this new methodological pipeline with the aim of exploring habitats that have low uncertainty (e.g., the initial ROIs) when calculating the ADC, with a higher signal-to-noise aiding more robust ADC quantification. This novel *Fit-Cluster-Fit* approach was used to improve the power of ADC values to discriminate benign and malignant neuroblastic tumors in children. This confidence habitats analysis was compared with two other methods: the standard voxel-based calculation of neuroblastic tumor aggressiveness in children; and a cluster-based strategy based on a voxel-wise cluster parcellation into two classes. If useful, this method will favor the use of DWI derived metrics as diagnostic oncological imaging biomarkers [8].

## 2. Materials and Methods

### 2.1. Patients and MR Images

The study included 33 consecutive pediatric oncology patients recruited from the hospital oncologic pediatric registry between 2010 and 2020. All patients had a pathological diagnosis of neuroblastic tumor after MR examination. The median age at first diagnosis was 23 months (mean 41 months, range 0–183 months), with no clear gender preference (female = 19; male = 14). The histopathological evaluation identified 21 children with neuroblastoma, 7 with ganglioneuroma and 5 with ganglioneuroblastoma. Each case was designated as benign (ganglioneuroma and intermixed ganglioneuroblastoma) or malignant (neuroblastoma and nodular ganglioneuroblastoma) [9]. Table 1 shows the descriptive statistics of the data set with respect to tumor malignancy.

Tumors were localized at different sites: cervical (*n* = 3), thoracic (*n* = 5), adrenal (*n* = 8), abdominal non-adrenal (*n* = 12), or pelvic (*n* = 5). Prognostic risk factors included the presence of metastases (*n* = 6), MYCN amplification (*n* = 2), ALK mutation (*n* = 3) and 11q deletion (*n* = 2). Information on the tumor stage at first diagnosis was retrieved from the electronic medical records (low risk, *n* = 20; intermediate risk, *n* = 5; and high risk, *n* = 7), indicating if it was a relapsing disease, and the therapeutic regime was noted.

All children underwent MRI examination under sedation with midazolam. MR images were acquired with a 1.5-T or 3-T scanner manufactured by either GEHC (*n* = 24), Siemens (*n* = 3) or Philips (*n* = 6). The MR protocol consisted of standard-of-care DWI, T1 and T2 weighted sequences with an in-plane spatial resolution ≤1 mm. Chest images were acquired with respiratory synchronization. The DW images were acquired with a fat saturated echo-planar imaging sequence having two *b*-values ranging from 0 to 1000 s/mm^2^.

The study was approved by the hospital’s Ethics Committee (The Ethics Committee for Investigation with medicinal products of the University and Polytechnic La Fe Hospital, ethic code: 2018/0228) and signed informed consent was waived due to the observational and retrospective study design.

### 2.2. Image Processing Methodology

The first step of the image processing pipeline consisted of applying the SUSAN noise filtering algorithm in order to reduce noise and preserve the underlying structures in DW images [10]. Manual tumor segmentation was then performed on the T2-weighted images by a pediatric radiologist with more than 20 years’ experience. The tumor ROIs extracted from in the T2-weighted images were summed to obtain the volumes of interest (VOIs) of the tumor.

The denoised DW images were registered to the T2-weighted images, using the registration frameworks of Elastix [11] and the Advanced Normalization Tools [12] to apply B-splines algorithms and Mutual Information as the main metric. The segmented regions were transferred to the registered parametric ADC maps obtained with the standard voxel-based mono-exponential signal decay model (Figure S1 in Supplementary Materials). All image processing steps were performed with the MATLAB [13] and Python [14] programming languages.

### 2.3. Voxel Uncertainty Estimation

A crucial step before applying the novel *Fit-Cluster-Fit* methodology is the estimation of the voxel uncertainty associated to the DW images. Tumors have heterogeneous structures with regions of similar signal behavior or characteristics, identified as habitats [15]. Some inner voxels may be at the boundaries between these regions and therefore, their signal properties might differ due to a partial volume effect. Also, high *b*-value images are characterized by lower signal-to-noise ratios, introducing higher uncertainties. These voxel uncertainties increase the variability when measuring the tumor ADC.

**Figure 2:**
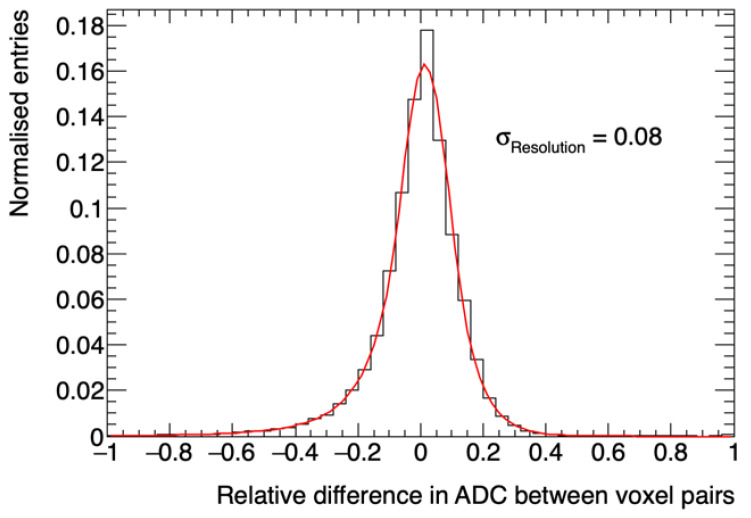
Relative difference in ADC between contiguous voxels calculated using a nearest-neighbor approach. The red line shows a double-sided Crystal Ball fit of the relative differences in ADC throughout the tumor.

By adopting a nearest neighbor approach, which assumes that the transition of intensities within a given tumor region is gradual, it was possible to attribute a value to these uncertainties. Our methodology identified the uncertainty per voxel by calculating the relative difference in ADC between each voxel and its eight nearest neighbors on a 2D plane (X-Y). Then, the relative differences in ADC were fitted to a double-sided Crystal Ball function, which is a probability density function consisting of a Gaussian core portion and two power-law tails below a certain threshold. The double-sided Crystal Ball fit is shown in Figure 2. A chi-squared goodness of fit was performed and found to be: χ^2^/ndf (number of degrees of freedom) = 295.5/41. The uncertainty per voxel was calculated considering the σ parameter, which determines the width of the Gaussian core with a value of 0.08.

### 2.4. The Fit-Cluster-Fit Method

**Figure 3:**
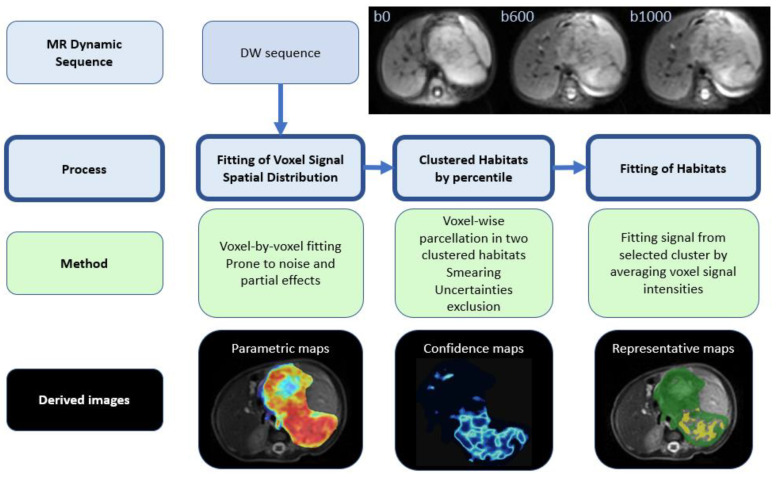
Scheme of the novel *Fit-Cluster-Fit* strategy. The following ranges were used: parametric maps, red (0.5·10^−3^ mm^2^/s)–blue (3.0·10^−3^ mm^2^/s); confidence maps, light blue (high voxel uncertainty)–dark blue (low voxel uncertainty); and representative maps, yellow (lower ADC habitat)–green (higher ADC habitat).

A novel automated methodology that is based on histogram analysis and clustering algorithms was developed and implemented to determine the representative tumor ADC. This methodology, referred to as *Fit-Cluster-Fit*, was designed to follow separate steps (Figure 3):

- Mono-exponential signal intensities were fit to each voxel within the tumor, obtaining the corresponding parametric voxel-based ADC maps [16].
- Single voxel assignation to one of two habitats (clusters) based on the percentiles. The optimal number of clusters (k = 2) was defined by performing a histogram analysis on the ADC distributions obtained for each case after applying the silhouette method [17] that maximizes the average silhouette over a range of possible k values. This step also included the identification and removal of voxels with high uncertainty, a strategy based on the calculation of confidence maps (further details below).
- The lower ADC habitat was selected, which is anticipated to correspond to the most aggressive regions of the tumor. Voxel signal intensities were averaged across this habitat and a second mono-exponential fit was performed considering the mean signal intensities per *b*-value within the selected habitat. A new visualization tool, called representative map, was developed to show the habitats identified.

The results from the *Fit-Cluster-Fit* methodology were compared to two other methods:

- Voxel-based (standard of care): Voxel-wise ADC maps were constructed by assuming a mono-exponential decay model. Mean ADCs for the whole tumor were calculated from the VOIs (voxel-by-voxel).
- Cluster-based: a voxel-wise cluster parcellation of the tumor into two classes was performed in order to identify two different habitats based on the histogram distribution of ADC. A no uncertainties exclusion was performed with this strategy and the lower ADC habitat was selected in all cases. The mean ADCs were calculated by averaging the ADC values (calculated voxel-by-voxel) within these habitats.

### 2.5. Cluster Definition: In Silico Experiments

The expected cluster assignment was obtained by smearing the data [18] with a double-sided Crystal Ball function to generate 100 ADC in silico experiments per case (Figure S2 in Supplementary Materials). The double-sided Crystal Ball function was chosen due to the need to have a consistent shape of the most optimal function found when fitting the voxel uncertainty histogram distribution (see Section 2.3 “*Voxel Uncertainty Estimation*”). The σ parameter chosen was equal to that obtained in the corresponding fit distribution to account for any statistical error in the measurement. The experiments were repeated 100 times to ensure that the confidence maps were accurate to a statistical uncertainty of less than 5%. The boundaries of the clusters were obtained based on the 5th, 10th and 15th percentile (independently) of the artificially generated ADC distributions.

### 2.6. Voxel Removal: Confidence Maps

The data from the computerized in silico experiments were used to calculate the accuracy of assigning a particular voxel to a cluster. This calculation was performed by estimating the frequency with which a voxel was assigned to one of the two defined clusters. The results are portrayed in confidence maps (Figure S3 in Supplementary Materials) and as expected, the voxels at the edge of a cluster have a lower habitat assignment accuracy.

The voxels with a low cluster assignment accuracy were removed based on a certainty metric ranging from 50% (where the voxel is assigned to each cluster the same number of times) to 95% (where the voxel is assigned to the same cluster in almost every experiment). Different threshold values (60%, 70%, 80%, 90% and 95%) were explored, and the optimal one was selected by minimizing false positive and false negative error probabilities, providing a balance between discrimination power and the removal of low accuracy voxels.

Once the clusters had been defined and the threshold for voxel removal established, in the final step of the *Fit-Cluster-Fit* method the average signal intensities per habitat were estimated to calculate the corresponding region-fit ADC value with the mono-exponential model (Figure S4 in Supplementary Materials). It is important to emphasize that the ADC calculations in the cluster-based and *Fit-Cluster-Fit* models were performed using the values from the lower ADC habitat, anticipated to correspond to most aggressive regions of the tumor. All ADC values were expressed as the mean and standard deviation of the corresponding parameter in each case (either signal intensity or ADC values—per voxel and/or within a cluster).

### 2.7. Statistical Analysis

A box-and-whisker plot was used to depict the distribution of the mean ADC values for benign and malignant neuroblastic tumor profiles in the voxel-based, cluster-based and *Fit-Cluster-Fit* models using different confidence thresholds to exclude uncertainties (60%, 70%, 80%, 90% and 95%). The optimal ADC threshold to differentiate malignant from benign neuroblastic tumors was determined using a receiver-operating characteristic (ROC) analysis. The accuracy, sensitivity, specificity, positive predictive value (PPV) and negative predictive value (NPV) of the methods explored were calculated for different parcellation thresholds, allowing voxel-wise clustering in two habitats. A cut-off value was determined to predict lesion malignancy based on the maximum value of the Youden index calculated for every point on the ROC curve (sensitivity  +  specificity  − 1).

Differences in the diagnostic performance of the clustered ADC values were analyzed by comparing the ROC curves. The discriminating power of each of the methods explored was evaluated through the difference in the ADC values between malignant and benign profiles using an unpaired two-tailed Student’s *t*-test. In recent years, there have been claims that the standards of evidence for new discoveries are simply too low in many scientific areas [19,20]. To improve reproducibility of scientific research, only *p*-values lower than 0.001 are considered as statistically significant.

The statistical analysis was performed using Python software [14] and the scikit-learn library [21].

## 3. Results

**Figure 4:**
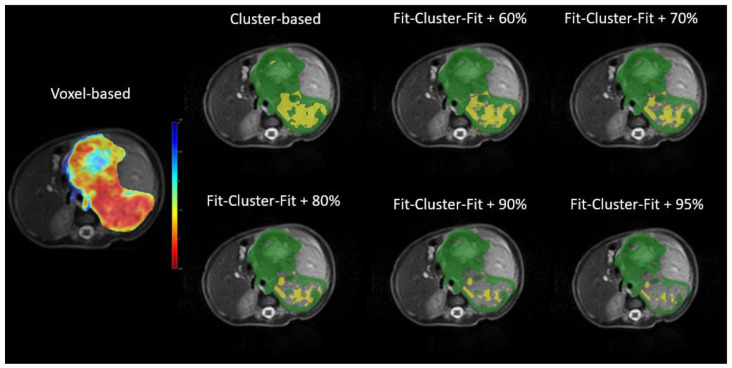
Parametric (**left**) and representative (**right**) maps of the apparent diffusion coefficient (ADC) in the voxel-based, cluster-based and *Fit-Cluster-Fit* models at different confidence thresholds for uncertainty exclusion (60%, 70%, 80%, 90% and 95%). The ranges established were: parametric maps, red (0.5·10^−3^ mm^2^/s)–blue (3.0·10^−3^ mm^2^/s); and representative maps, yellow (lower ADC habitat)–green (higher ADC habitat).

Representative maps for the ADC values in the voxel-based, cluster-based and *Fit-Cluster-Fit* models are shown in Figure 4 for the different confidence thresholds to exclude uncertainties (60%, 70%, 80%, 90% and 95%). Those voxels with low ADC values in the parametric map were mostly assigned to the lower ADC habitat in the respective representative maps. As the exclusion threshold increased, more high-uncertainty voxels were removed from their corresponding cluster, with the extreme scenario of encountering a few low-uncertainty voxels belonging to the most aggressive habitat.

When evaluating the power of the mean ADC to differentiate malignant and benign neuroblastic tumors, all the different combinations of the cluster-based and *Fit-Cluster-Fit* models produced statistically significant differences (*t*-test), with small discrepancies in their differentiation power. The nominal *Fit-Cluster-Fit* method (with no uncertainty exclusion), showed a *p*-value of 0.0005 and an effect size (Cohen’s d) of 2.69. By contrast, the standard voxel-based method produced no significant differences between benign and malignant neuroblastic tumors in terms of the mean ADC values (*p* = 0.0166, Cohen’s d = 1.44). Therefore, the cluster-based and *Fit-Cluster-Fit* models produced a significant improvement in the tumor differentiation power compared to the voxel-based standard-of-care method.

**Figure 5:**
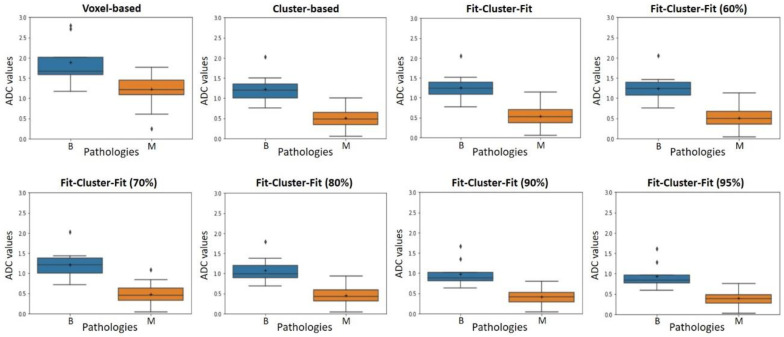
Box-and-whisker plot representations of the apparent diffusion coefficient (ADC, 10^−3^ mm^2^/s) for benign (B) and malignant (M) neuroblastic tumors in voxel-based, cluster-based and *Fit-Cluster-Fit* models, applying different confidence thresholds to exclude uncertainties (60%, 70%, 80%, 90% and 95%). Outliers are plotted as individual points.

The box-and-whisker plots (Figure 5) show the distribution of the mean ADC values for benign and malignant neuroblastic tumors. These results are in agreement with the *p*-values obtained from the *t*-test, as the cluster-based and *Fit-Cluster-Fit* models showed a strong and similar distinction between benign and malignant tumors, as opposed to the voxel-based method. As a general trend, the interquartile ranges were lower with the *Fit-Cluster-Fit* methodologies when the confidence threshold to exclude uncertainties increased.

### 3.1. Optimal ADC Threshold per Method and Evaluation of the Performance Metrics

The detailed data per tumor malignancy profile for the various voxel-based, cluster-based and *Fit-Cluster-Fit* strategies is summarized in Table 2, with the different confidence thresholds to exclude uncertainties (60%, 70%, 80%, 90% and 95%) according to the ADC cut-off value within a cluster. The corresponding mean ADC value, sensitivity, specificity, accuracy, PPV, NPV and AUC-ROC values are shown.

The ADC cut-off value for each method was determined according to the Youden index. The use of the *Fit-Cluster-Fit* method applied in conjunction with the strategy to exclude uncertainties at a confidence threshold of 70% had a higher sensitivity (91%), NPV (80%) and accuracy (91%) for the profiles of malignancy than the rest of the combinations and methods explored with a similarly high specificity (89%) and PPV (95%). For instance, a lower sensitivity (87%) and NPV (75%) was associated with the cluster-based method, yet with a higher specificity (100%) and PPV (100%) for the malignancy profiles. The voxel-based strategy had a lower sensitivity (87%), NPV (70%) and accuracy (84%), which was associated with a considerable decrease in its specificity (78%). Therefore, the 70% threshold *Fit-Cluster-Fit* approach improved the sensitivity and NPV by 4% and 10%, respectively, relative to the voxel-based method, and by 4% and 5%, respectively, relative to the cluster-based method.

**Figure 6:**
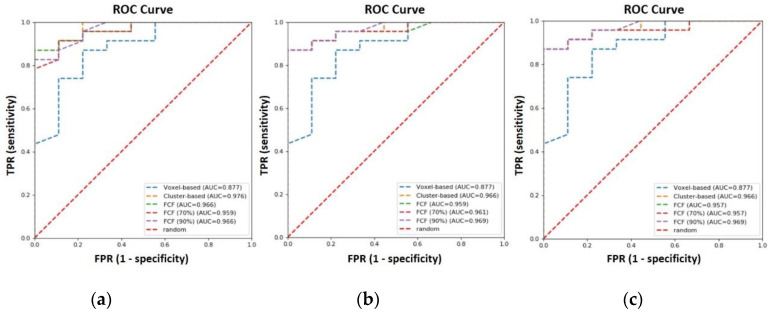
Receiver-operating characteristic (ROC) curves for voxel-based, cluster-based and *Fit-Cluster-Fit* models when applying different confidence thresholds (70% and 90%) to exclude uncertainties, and after performing a voxel-wise clusterization into two habitats with the (**a**) 5th, (**b**) 10th and (**c**) 15th percentiles as the parcellation thresholds.

The ROC curves for the cluster-based and *Fit-Cluster-Fit* methods were obtained after performing a voxel-wise clustering into two habitats with the 5th, 10th and 15th percentiles as parcellation thresholds (Figure 6), including the standard voxel-based strategy for comparison. 

Of all the ROC curves generated, the cluster-based model has the highest AUC (0.976), followed by the Fit-Cluster-Fit strategy with exclusion of uncertainties at 80% and 90% confidence level. An AUC of 0.967 was obtained when this correction was not applied. For completeness, it should be noted that the remaining Fit-Cluster-Fit models have a very similar AUC. For instance, the corresponding models gave an AUC of 0.964, 0.961 and 0.959 at a confidence threshold of 95%, 60% and 70%, respectively. The largest difference in AUC was achieved with the voxel-based methodology that produced an AUC of 0.877.

### 3.2. Parcellation Threshold to Define Habitats

Modifying the parcellation threshold to the 10th and 15th percentiles for the voxel-wise clustering into two habitats did not improve the performance obtained with the *Fit-Cluster-Fit* model at a 70% confidence threshold when using the 5th percentile as the threshold for parcellation. Considering the voxel-based, cluster-based and *Fit-Cluster-Fit* methods, the highest values of sensitivity and NPV to distinguish the profiles of malignancy were 87% and 75%, respectively, obtained when using both the 10th and 15th percentile configurations (Tables S1 and S2 in Supplementary Materials). Therefore, by using the 5th percentile as the parcellation threshold, the sensitivity and the NPV to distinguish malignancy profiles increases by 4% and 5%, respectively.

### 3.3. Multivendor Variability

To evaluate the robustness of the methods developed in terms of the variability introduced by the multivendor image data used in this study, a Tukey HSD test was applied for multiple comparisons of the ADC means. There were no significant differences in the results obtained with the *Fit-Cluster-Fit* method combined with the 70% uncertainty exclusion technique between the different vendors in terms of the mean ADC values for each tumor malignancy (GE-Siemens *p* = 0.68, GE-Philips *p* = 0.31, Siemens-Philips *p* = 0.25). To evaluate the variability associated with the main magnetic field strength of the scanner, an unpaired *t*-test was used to compare the ADC means. There were no significant differences between the two magnetic field strengths of the scanners (1.5–3 T) when the *Fit-Cluster-Fit* methodology was combined with the 70% exclusion of uncertainty (*p* = 0.13).

## 4. Discussion

The results obtained in this study show that benign and malignant neuroblastic tumor profiles can be successful differentiated in cluster-based and *Fit-Cluster-Fit* models when using the values from the lower ADC habitat. In particular, removing uncertainties at a 70% confidence threshold produced the best sensitivity (91%) and specificity (89%) of all the combinations and methods explored, improving standard voxel-based [3,4,5] sensitivity and NPVs by 4% and 10%, respectively. This improvement may be explained by the solutions adopted to overcome some of the different factors that introduce bias in the voxel-based approach, such as the low signal-to-noise ratio of the source pixels, the signal uncertainty due to partial volume effects, and the inaccurate representation of average values from tumors with a heterogeneous composition.

An alternative approach that has also been explored in recent years involves using different sized regions with similar signal intensities to calculate this parameter from the average signal [22]. Comparisons were performed to evaluate which measurement best predicted the tumor response to treatment when the ADC was measured over a larger area. This approach enables higher signal to noise ratios to be obtained and more robust signal fitting for a defined region. However, this approach also introduces considerable variability in the delineation of the region studied by different operators.

Previous studies have identified tumor habitats based on the voxel signal intensities of several MR sequences (fluid attenuated inversion recovery, T1-weighted, post-contrast T1-weighted and T2-weighted) or modalities (PET/CT), and the results have been validated by extracting radiomic features from each cluster to test their predictive power against a specific clinical outcome [23,24]. Nevertheless, the out-of-the-box clustering algorithms employed in these studies usually produce large numbers of tumor clusters that are not generally well justified from a physiological perspective. In addition, the conclusions obtained from this type of analysis are very dependent on the clinical outcome under study. For instance, the tumor habitats defined in a particular scenario might not be suitable to another situation.

As a result, here we designed a novel methodology to deal with these limitations. To overcome the problem of high tumor variability and the lack of generalizability, voxel uncertainty is quantified and voxels with lower confidence values are excluded, resulting in a selection of more homogeneous and representative tumor habitats that are independent of the problem under study. Moreover, and taking advantage of the results obtained in previous studies on ADC measurement techniques [25], the signal intensities of the voxels belonging to the lower ADC habitat are averaged and fitted in order to evaluate the robustness of performing a second fit on the signal intensities rather than averaging the voxel-based ADC values within these habitats.

DW images have also been used previously to define tumor habitats, such as for prostate cancer or soft-tissue sarcoma [26,27]. In this case, clustering was performed through a 2-step approach, whereas the *Fit-Cluster-Fit* can be considered as a 3-step approach in which the uncertainty of the imaging biomarker under study (ADC) is further estimated at a voxel level and used to improve clustering. Multi-habitat analysis of tumors not-specific to DW signals have been proposed elsewhere, combining this as a post-processing step to obtain prognostic models of treatment response or of the clinical significance of lesions [28,29]. Our approach can also be applied to evaluate treatment response by analyzing the longitudinal changes of the clusters after treatment cycles, both in terms of the size and characteristics of the histogram.

Improved differentiation between benign and malignant profiles of neuroblastic tumors was observed by adopting a univariate approach to examine the ADC relative to standard voxel-based methods. The results highlight the benefits of performing a voxel-wise cluster parcellation into two classes, incorporating a smearing process to more accurately predict the expected cluster assignment with less than 5% statistical error, also applying an uncertainty exclusion strategy to identify and remove noise and poor-quality lesion voxels.

It is noteworthy that there are few tools to visualize tumor uncertainty, and that may be useful to provide insights into the reproducibility and accuracy of the measurements. Therefore, three visualization algorithms were developed to account for the different symbolic representations of tumor heterogeneity: parametric maps, confidence maps and representative maps. The confidence maps obtained represent the first attempt to assign uncertainty to the voxels within a specific tumor habitat. The combination of these representations with other sources of data, such as genomic, molecular and biological data, paves the way for new insights into tumor heterogeneity based on the more accurate definition of habitats. This has the potential to drive more personalized diagnosis, prognosis and response evaluation of patients with diverse tumors. By following this strategy, it may be possible to explore the different cluster models developed in this study, applying multivariate approaches to validate the *Fit-Cluster-Fit* methodology, and to evaluate the correlation and significance of the multi-disciplinary data in a multivariate regression environment. This same *Fit-Cluster-Fit* approach can be applied to more complex signal analysis if DW images are acquired with more *b*-values, such as the Intravoxel Incoherent Motion, the Stretched-exponential and the Diffusion Kurtosis models. These analyses provide further metrics, although there is a generally poor consensus regarding the balance between signal quality, spatial resolution and acquisition time that limits their implementation.

A limitation of the study is that it was conducted at a single institution and that a relatively low number of cases were included. Additional studies on larger cohorts from different centers will be necessary to validate our data, and to optimize the cluster-based and *Fit-Cluster-Fit* models. Also, the influence of different image preparation processes should be further evaluated, particularly in terms of the harmonization of multivendor imaging data. In this sense, there were no significance differences in the results obtained with the *Fit-Cluster-Fit* method between the distinct vendors (GE, Philips, Siemens) and magnetic field strengths of the scanners (1.5–3 T), indicating that the strategy is more robust against variability in data acquisition than other state-of-the-art solutions [30]. An interesting benchmark test to compare different *Fit-Cluster-Fit* approaches is the creation of ad-hoc digital reference objects with no gradient variation of the parameter under study (such as the ADC) but with the values arranged in a cluster-like structures.

## 5. Conclusions

The *Fit-Cluster-Fit* method presented here improves the classification performance of imaging biomarkers for pediatric solid tumor cancers and it can be adapted to evaluate any dynamic tumor signal.

## Figures and Tables

**Table 1:** Descriptive statistics of the cohort in terms of tumor malignancy. The cohort includes 33 consecutive pediatric oncology patients in the hospital oncological register from 2010 to 2020.

Statistics of the Data Set		Benign Neuroblastic Tumors (*n* = 10)	Malignant Neuroblastic Tumors (*n* = 23)	All Tumors
				(*n* = 33)
Age at first diagnosis (months)	Mean	73	27	41
	Median	77	17	23
	Standard Deviation	5	3	4
Gender, *n* (%)	Male	2 (6)	12 (36)	14 (42)
	Female	8 (24)	11 (33)	19 (58)
Tumor location, *n* (%)	Cervical	1 (3)	2 (6)	3 (9)
	Thoracic	2 (6)	3 (9)	5 (15)
	Abdominal (adrenal)	3 (9)	5 (15)	8 (24)
	Abdominal (non-adrenal)	2 (6)	10 (30)	12 (36)
	Pelvic	2 (6)	3 (9)	5 (15)
Tumor stage at first diagnosis, *n* (%)	Low risk	9 (27)	11 (33)	20 (61)
	Intermediate risk	1 (3)	4 (12)	5 (15)
	High risk	0 (0)	7 (21)	7 (21)
Prognostic risk factors, *n* (%)	Metastases	0 (0)	6 (18)	6 (18)
	MYCN amplification	0 (0)	2 (6)	2 (6)
	ALK mutation	0 (0)	3 (9)	3 (9)
	11q deletion	0 (0)	2 (6)	2 (6)
Manufacturer (MR), *n* (%)	GE	9 (27)	15 (45)	24 (73)
	Philips	0 (0)	6 (18)	6 (18)
	Siemens	1 (3)	2 (6)	3 (9)
MR Field strength, *n* (%)	1.5 T	6 (18)	17 (52)	23 (70)
	3 T	4 (12)	6 (18)	10 (30)

**Table 2:** The apparent diffusion coefficient (ADC) according to tumor malignancy in the voxel-based, cluster-based and *Fit-Cluster-Fit* models, applying different confidence thresholds to exclude uncertainties (60%, 70%, 80%, 90% and 95%). Clustered habitats were built based on the 5th percentile of the artificially generated ADC distributions.

Method	ADC Cut-Off	ADC Value (10^−3^ mm^2^/s)		Sensitivity (%)	Specificity (%)	Accuracy (%)	PPV (%)	NPV (%)	AUC
		Benign	Malignant						
Voxel-based	1.56	1.89 ± 0.55	1.23 ± 0.34	87	78	84	91	70	0.877
Cluster-based	0.75	1.22 ± 0.38	0.50 ± 0.23	87	100	91	100	75	0.976
FCF	0.77	1.25 ± 0.38	0.53 ± 0.24	87	100	91	100	75	0.967
FCF (60%)	0.75	1.24 ± 0.38	0.51 ± 0.24	83	100	88	100	69	0.961
FCF (70%)	0.77	1.21 ± 0.39	0.48 ± 0.24	91	89	91	95	80	0.959
FCF (80%)	0.68	1.07 ± 0.34	0.45 ± 0.22	87	100	91	100	75	0.967
FCF (90%)	0.60	0.98 ± 0.33	0.41 ± 0.20	83	100	88	100	69	0.967
FCF (95%)	0.57	0.94 ± 0.32	0.39 ± 0.19	83	100	88	100	69	0.964

## Supplementary Materials

The following are available online at https://www.mdpi.com/2072-6694/12/12/3858/s1, Figure S1: (**a**) Parametric map and (**b**) distribution histogram of the apparent diffusion coefficient (ADC, 10^−3^ mm^2^/s) obtained with the standard voxel-based mono-exponential signal decay model. Figure S2: Generation of *in-silico* experiments, smearing of ADC values with a double-sided Crystal Ball function and cluster assignment of voxels. At the bottom, frequency map and frequency distribution of voxel allocation. Figure S3: (**a**) Confidence map obtained from in-silico experiments representing the accuracy of voxel allocation to a clustered habitat. (**b**) The distribution of the confidence level spectrum per voxel can be used to threshold the voxel groups to exclude uncertainties. Figure S4: (**a**) Apparent diffusion coefficient (ADC) map (10^−3^ mm^2^/s) obtained with the *Fit-Cluster-Fit* method. (**b**) Distribution of the ADC spectrum performing a voxel-wise clustering into two habitats, with the 5th percentile as the parcellation threshold. Table S1: Apparent diffusion coefficient (ADC) values per tumor malignancy in voxel-based, cluster-based and *Fit-Cluster-Fit* models with different confidence thresholds to exclude uncertainties (60%, 70%, 80%, 90% and 95%). Clustered habitats were built based on the 10th percentile of the artificially generated ADC distributions. Table S2: Apparent diffusion coefficient (ADC) values per tumor malignancy in voxel-based, cluster-based and *Fit-Cluster-Fit* models with different confidence thresholds to exclude uncertainties (60%, 70%, 80%, 90% and 95%). Clustered habitats were built based on the 15th percentile of the artificially generated ADC distributions.

## Abbreviations

ADC	Apparent Diffusion Coefficient
AUC	Area under the curve
DWI	Diffusion-weighted imaging
EPI	echo-planar imaging
HSD	Honestly significant difference
mIBG	Metaiodobenzylguanidine scintigraphy
MR	Magnetic Resonance
ndf	Number of degrees of freedom
NPV	negative predictive value
PPV	positive predictive value
ROC	Receiver operating characteristic
ROI	Region of interest
SSFSE	Single shot fast spin echo
STIR	Short-tau inversion-recovery
VOI	Volume of interest
