# Supplementary material for: A Confidence Habitats Methodology in MR Quantitative Diffusion for the Classification of Neuroblastic Tumors
Source: Cancers (Basel). 2020 Dec 21;12(12):3858. doi: 10.3390/cancers12123858 (PMC7767170; doi:10.3390/cancers12123858)
Supplement: Supplementary file 1 [file cancers-12-03858-s001.pdf]

cancers

MDPI

Supplementary Materials

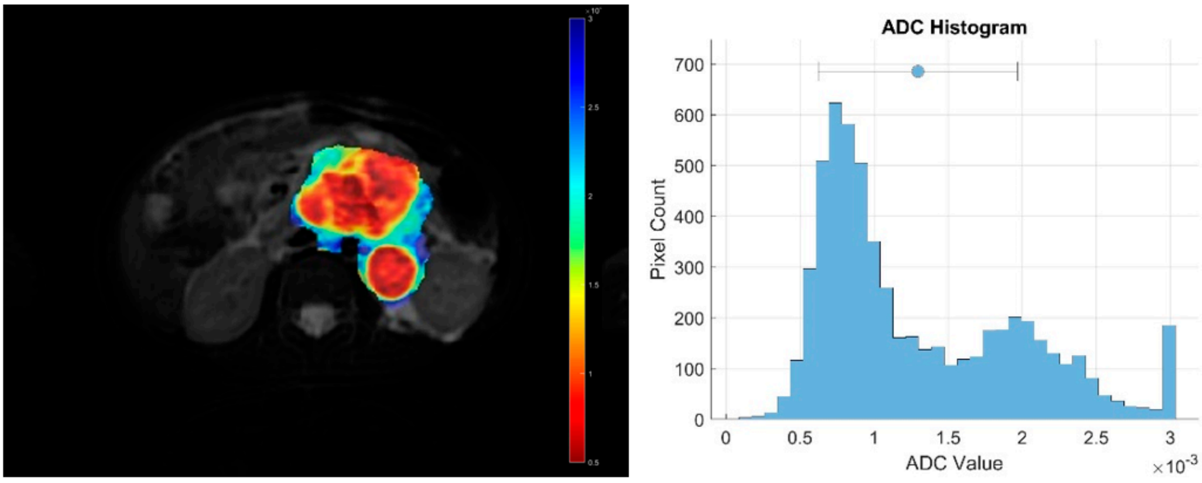

(a) (b)

Figure S1. (**a**) Parametric map and (**b**) distribution histogram of the apparent diffusion coefficient (ADC, 10^{-3} mm^2/s) obtained with the standard voxel-based mono-exponential signal decay model.

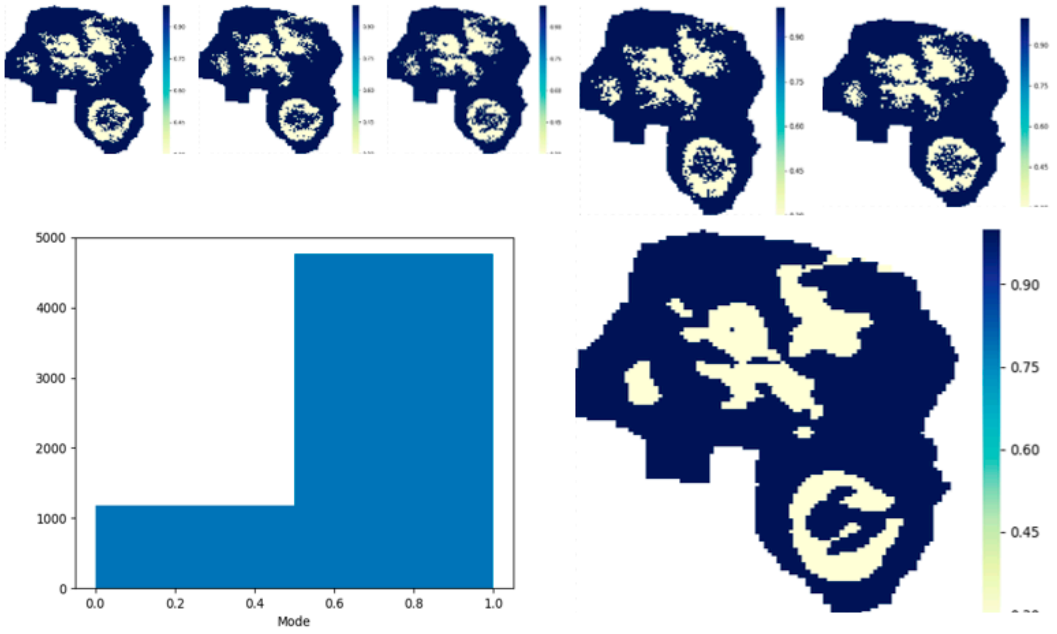

Figure S2. Generation of *in-silico* experiments, smearing of ADC values with a double-sided Crystal Ball function and cluster assignment of voxels. At the bottom, frequency map and frequency distribution of voxel allocation.

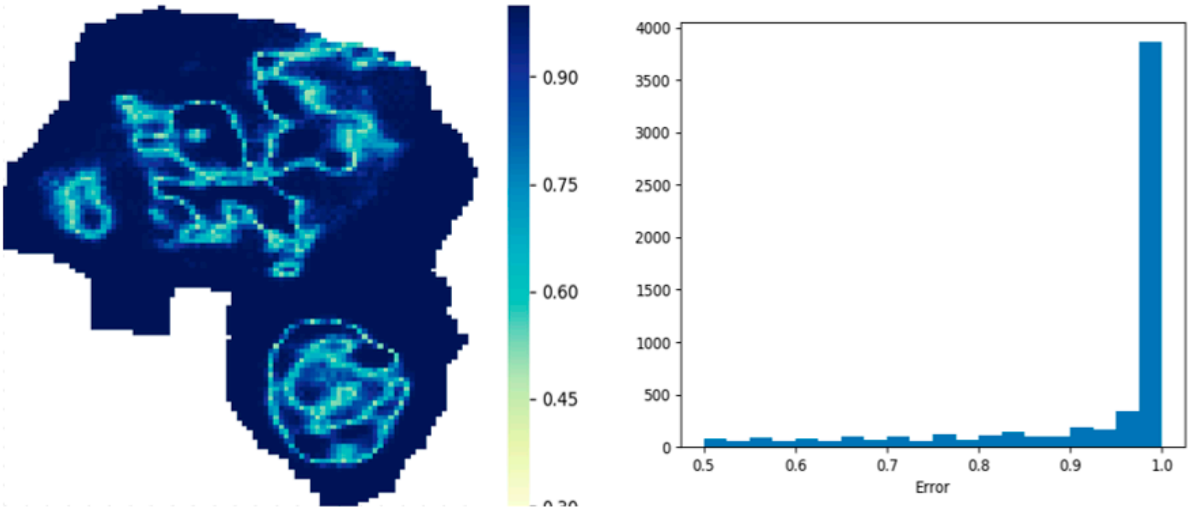

(**a**) (**b**)

Figure S3. (**a**) Confidence map obtained from in-silico experiments representing the accuracy of voxel allocation to a clustered habitat. (**b**) The distribution of the confidence level spectrum per voxel can be used to threshold the voxel groups to exclude uncertainties.

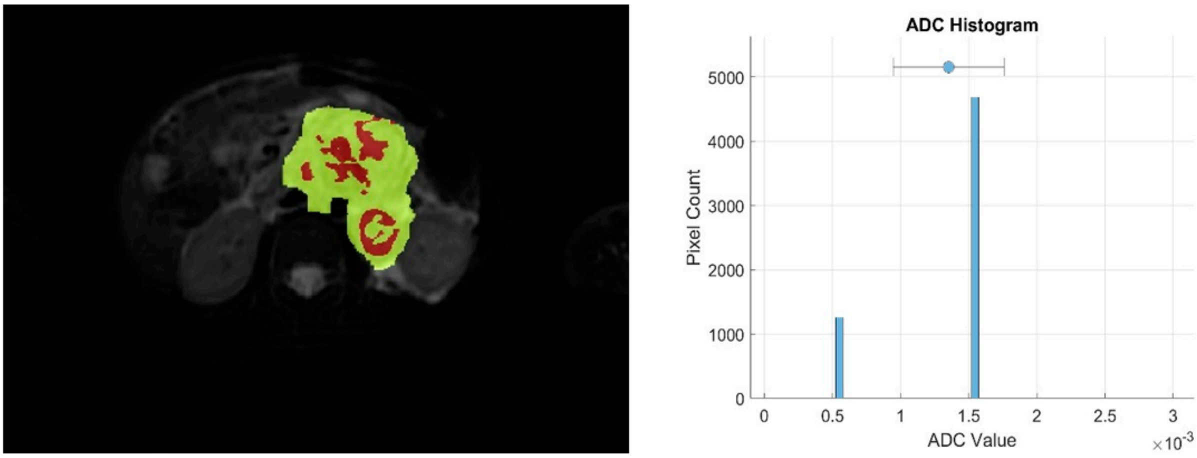

(**a**) (**b**)

Figure S4. (**a**) Apparent diffusion coefficient (ADC) map (10^{-3} mm^2/s) obtained with the *Fit-Cluster-Fit* method. (**b**) Distribution of the ADC spectrum performing a voxel-wise clustering into two habitats, with the 5th percentile as the parcellation threshold.

Table S1. Apparent diffusion coefficient (ADC) values per tumor malignancy in voxel-based, cluster-based and *Fit-Cluster-Fit* models with different confidence thresholds to exclude uncertainties (60%, 70%, 80%, 90% and 95%). Clustered habitats were built based on the 10th percentile of the artificially generated ADC distributions.

Method	ADC cut-off value	ADC value (10^{-3} mm^2/s)		Sensitivity (%)	Specificity (%)	Accuracy (%)	PPV (%)	NPV (%)	AUC
		Benign	Malignant						
Voxel-based	1.56	1.89±0.55	1.23±0.34	87	78	84	91	70	0.877
Cluster-based	0.80	1.33±0.41	0.60±0.24	87	100	91	100	75	0.966
FCF	0.81	1.36±0.42	0.63±0.24	87	100	91	100	75	0.959
FCF (60%)	0.76	1.33±0.41	0.61±0.24	83	100	88	100	69	0.959
FCF (70%)	0.76	1.27±0.40	0.58±0.24	87	100	91	100	75	0.961
FCF (80%)	0.71	1.18±0.36	0.54±0.21	87	100	91	100	75	0.966
FCF (90%)	0.69	1.11±0.35	0.51±0.20	87	100	91	100	75	0.969
FCF (95%)	0.68	1.06±0.35	0.49±0.19	87	100	91	100	75	0.966

Table S2. Apparent diffusion coefficient (ADC) values per tumor malignancy in voxel-based, cluster-based and *Fit-Cluster-Fit* models with different confidence thresholds to exclude uncertainties (60%, 70%, 80%, 90% and 95%). Clustered habitats were built based on the 15th percentile of the artificially generated ADC distributions.

Method	ADC cut-off value	ADC value (10^{-3} mm^2/s)		Sensitivity (%)	Specificity (%)	Accuracy (%)	PPV (%)	NPV (%)	AUC
		Benign	Malignant						
Voxel-based	1.56	1.89±0.55	1.23±0.34	87	78	84	91	70	0.877
Cluster-based	0.83	1.12±0.52	0.60±0.24	87	100	91	100	75	0.966
FCF	0.83	1.13±0.57	0.63±0.24	87	100	91	100	75	0.957
FCF (60%)	0.82	1.10±0.57	0.61±0.24	87	100	91	100	75	0.957
FCF (70%)	0.77	1.05±0.55	0.58±0.24	87	100	91	100	75	0.957
FCF (80%)	0.75	0.96±0.49	0.54±0.21	87	100	91	100	75	0.966
FCF (90%)	0.72	0.91±0.47	0.51±0.20	87	100	91	100	75	0.969
FCF (95%)	0.70	0.87±0.45	0.49±0.19	87	100	91	100	75	0.971
